# Optimized submerged batch fermentation for metabolic switching in *Streptomyces yanglinensis* 3–10 providing platform for reveromycin A and B biosynthesis, engineering, and production

**DOI:** 10.3389/fmicb.2024.1378834

**Published:** 2024-05-07

**Authors:** Longyan Yang, Qaiser Shakeel, Xueqin Xu, Liaqat Ali, Zhiyan Chen, Mustansar Mubeen, Muhammad Aamir Sohail, Yasir IfItikhar, Ajay Kumar, Manoj Kumar Solanki, Yun Zhou, Dongling Zhao, Nada K. Alharbi, Jie Wang

**Affiliations:** ^1^China Tobacco Guangxi Industrial Co., Ltd., Nanning, China; ^2^Cholistan Institute of Desert Studies, Faculty of Agriculture and Environment, The Islamia University of Bahawalpur, Bahawalpur, Pakistan; ^3^Department of Plant Pathology, College of Agriculture, University of Sargodha, Sargodha, Pakistan; ^4^National Key Laboratory of Plant Molecular Genetics, Center for Excellence in Molecular Plant Sciences, Institute of Plant Physiology and Ecology, Chinese Academy of Sciences, Shanghai, China; ^5^Amity Institute of Biotechnology, Amity University, Noida, Uttar Pradesh, India; ^6^Department of Life Sciences and Biological Sciences, IES University, Bhopal, Madhya Pradesh, India; ^7^Plant Cytogenetics and Molecular Biology Group, Institute of Biology, Biotechnology and Environmental Protection, Faculty of Natural Sciences, University of Silesia in Katowice, Katowice, Poland; ^8^Department of Biology, College of Science, Princess Nourah bint Abdulrahman University, Riyadh, Saudi Arabia; ^9^Tobacco Research Institute, Chinese Academy of Agricultural Sciences, Qingdao, China

**Keywords:** *Streptomyces*, submerged fermentation, optimization, antimicrobial activity, biocontrol

## Abstract

The cultivation system requires that the approach providing biomass for all types of metabolic analysis is of excellent quality and reliability. This study was conducted to enhance the efficiency and yield of antifungal substance (AFS) production in *Streptomyces yanglinensis* 3–10 by optimizing operation conditions of aeration, agitation, carbon source, and incubation time in a fermenter. Dissolved oxygen (DO) and pH were found to play significant roles in AFS production. The optimum pH for the production of AFS in *S. yanglinensis* 3–10 was found to be 6.5. As the AFS synthesis is generally thought to be an aerobic process, DO plays a significant role. The synthesis of bioactive compounds can vary depending on how DO affects growth rate. This study validates that the high growth rate and antifungal activity required a minimum DO concentration of approximately 20% saturation. The DO supply in a fermenter can be raised once agitation and aeration have been adjusted. Consequently, DO can stimulate the development of bacteria and enzyme production. A large shearing effect could result from the extreme agitation, harming the cell and deactivating its products. The highest inhibition zone diameter (IZD) was obtained with 3% starch, making starch a more efficient carbon source than glucose. Temperature is another important factor affecting AFS production. The needed fermentation time would increase and AFS production would be reduced by the too-low operating temperature. Furthermore, large-scale fermenters are challenging to manage at temperatures that are far below from room temperature. According to this research, 28°C is the ideal temperature for the fermentation of *S. yanglinensis* 3–10. The current study deals with the optimization of submerged batch fermentation involving the modification of operation conditions to effectively enhance the efficiency and yield of AFS production in *S. yanglinensis* 3–10.

## 1 Introduction

*Streptomyces* species is filamentous, soil-dwelling bacteria renowned for their potential for secondary metabolite production, such as antifungal substances, antibiotics, immunosuppressant, anticancer compounds, and several other bioactive molecules (Chen et al., 2016; Quinn et al., 2020; Barbuto Ferraiuolo et al., 2021). *Streptomyces* species produce over 75% of bioactive compounds (Demain, 2000). Consequently, *Streptomyces* species have been studied thoroughly as biocontrol agents to manage several phytopathogens. Efforts for the application of *Streptomyces* species against phytopathogens were tracked down when in 1927, it was discovered that *Streptomyces praecox* efficiently reduced potato scab disease upon its soil application either alone or in combination with green manure (Millard and Taylor, 1927). *S. griseus* produces streptomycin which effectively suppresses *Erwinia amylovora* causing pear fire blight. This was the first time when an antibiotic produced by *Streptomyces* was used against any plant disease (Beer et al., 1984). Since then, a lot of study has been conducted to use *Streptomyces* as biofungicides or fertilizers. Several *Streptomyces* species, including Rhizovit R and Mycostop R, have successfully developed into commercial bio-fungicides due to their viable spores or bioactive chemicals (Berg et al., 2001; Minuto et al., 2006) or kasugamycin, blasticidin, validamycins, and polyoxins (Kim and Hwang, 2007). Considering the success of such applications, determining new strains of *Streptomyces* and finding novel antibiotics produced by *Streptomyces* species remains a popular and lucrative field of research. Many new antibiotics are being evaluated as potential biofungicides, such as the novonestmycins isolated from *S. phytohabitans* (Wan et al., 2015), bafilomycin K isolated from *S. flavotricini* Y12-26 (Zhang et al., 2011), and elaiomycins B isolated from *Streptomyces* sp. BK190 (Kim et al., 2011). The volatile organic compounds of *S. yanglinensis* 3–10 can successfully inhibit the growth of *Aspergillus parasiticus* and *A. flavus* and the generation of aflatoxin in *A. parasiticus*, according to an *in vitro* investigation. It has also been revealed that the antifungal substances produced by *S. yanglinensis* 3–10 are effective against the growth of *A. flavus* and AFB_1_ aflatoxin production under both *in vitro* and *in vivo* settings (Shakeel et al., 2018).

Two compounds produced by *S. yanglinensis* 3–10 were purified from the crude extract and were identified as reveromycins A and B, which demonstrated high antifungal activity against *Botrytis cinerea, Mucor hiemalis, Rhizopus stolonifer,* and *Sclerotinia sclerotiorum* under acidic pH conditions (Lyu et al., 2017). Due to the production of reveromycins, *Streptomyces* species has become an increasingly intriguing topic in recent years. Reveromycins are the first antibiotics identified in *Streptomyces reveromyceticus* SN-593 (Osada et al., 1991). Reveromycin A is a substance with a variety of biological properties (Osada, 2016). Its capacity to inhibit the proliferation of cells triggered by the epidermal growth factor has been investigated, and this might be significant in the treatment of cancer. It can also lessen tumors that are hormone-influenced, such as those present in ovarian and prostate cancer (Osada et al., 1991; Takahashi et al., 1997). Furthermore, Reveromycin A has been shown to trigger a process called apoptosis in osteoclasts, which could help in reducing fractures (Takahashi et al., 1992; Woo et al., 2006; Fremlin et al., 2011).

However, the limited production yield of these bioactive compounds poses a challenge to their commercial viability. Therefore, it is essential to optimize the fermentation process of *S. yanglinensis* 3–10 to increase the production of these compounds and enhance their potential as commercial antifungal agents. To achieve this, the culture media and conditions required for the production of antifungal compounds by *S. yanglinensis* 3–10 have been carefully optimized. This optimization has not only increased the production of reveromycin but has also been effective against club root disease in oilseed rape (Shakeel et al., 2016). To consistently produce high-quality biomass for metabolic evaluation, an efficient cultivation strategy is crucial. Submerged batch fermentation has emerged as a popular technique for cultivating *Streptomyces* species, significantly boosting the production of bioactive compounds. This optimization process involves adjusting various factors, such as fermentation conditions, medium composition, and even genetic engineering, to maximize the yield of the desired compounds (Song et al., 2019; Zhang et al., 2019).

It is worth noting that further enhancement in both the quantity and quality of reveromycin production may be achieved by employing a combination of strategies, including medium optimization, fermentation condition manipulation, and genetic engineering techniques. This study aims to identify the optimal combination of aeration, agitation, carbon source, and incubation time to maximize the efficiency and yield of antifungal substances (AFSs), particularly reveromycin A and B, produced by *S. yanglinensis* 3–10 in a fermenter.

## 2 Materials and methods

### 2.1 Microbial strains

Two microbial strains, *S. yanglinensis* 3–10 and *Aspergillus niger* A-1, were used in this study. *S. yanglinensis* 3–10 was a mutant of isolate F-1 of *S. platensis* (Che, 2010). *S. yanglinensis* 3–10 was originally isolated from a healthy rice leaf grown in the field near Wuhan, China (Wan et al., 2008) and stored at −20°C. It was cultured on fermentation medium at 28°C for 72 h for AFS production. Cultural medium used for *S. yanglinensis* 3–10 was optimized (OPM) by Shakeel et al. (2016) which contains soluble starch 3%, peptone 0.75%, yeast extract 0.025%, soybean meal 1%, K_2_HPO_4_•3H_2_O 0.5 g/L, KH_2_PO_4_ 0.7 g/L, MgSO_4_·7H_2_O 0.4 g/L, MnSO_4_·H_2_O 0.02 g/L, and ZnSO_4_·7H_2_O 0.01 g/L. The medium ISP-2 contained (in 1,000 mL water) 4 g of yeast extract, 4 g of D-glucose, and 10 g of malt extract, pH 7 (Shirling and Gottlieb, 1966). It was used as a seed medium for *S. yanglinensis* 3–10. Potato dextrose agar (PDA) was prepared with peeled potato tubers using the procedures described by Fang (1998). It was used in bioassays to test the antifungal activity of cultural filtrates of *S. yanglinensis* 3–10. In our previous study*, A. niger* A-1 was isolated from a decayed sclerotium of *Sclerotinia sclerotiorum* (Lu, 2010; Shakeel et al., 2016). It was used as an indicator in bioassays to detect the antifungal activity of the cultural filtrates of *S. yanglinensis* 3–10.

### 2.2 Preservation and revival of strains

Spore batches were produced by cultivating spores of *S. yanglinensis* 3–10 on a fermentation medium (Shakeel et al., 2016), while *A. niger* A-1 on potato dextrose agar plates (Dynowska et al., 2011) harvested spores by scraping and individually suspending them in 20% glycerol. The spore suspensions of both strains were then individually stored as glycerol stock at −80°C by following the method described by Shepherd et al. (2010). The strains were re-inoculated on freshly prepared respective medium with the help of a sterile loop and were incubated at 28 ± 2°C for 7 days as described by Saxena and Gupta (2019).

### 2.3 Antifungal assay

The AFS bioassays were carried out in square-shaped plates (260 × 215 × 20 mm, length × width × depth), each containing 180 mL PDA and 20 mL conidial suspension of *A. niger* (1 × 10^8^ conidia/ml). Sterilized stainless-steel Oxford cups (10 × 6 × 8 mm, height × inner diameter × outer diameter) were placed on the surface of the PDA plates. Three aliquots (200 μL) of each cultural filtrate from each culture of *S. yanglinensis* 3–10 were pipetted in the three cups, respectively. The plates were placed in an incubator at 37°C for 72 h, and the diameter of the clear zones around each cup was measured (Shakeel et al., 2016).

### 2.4 Inoculum preparation

The inoculum was prepared by slightly modifying the procedure described by Lyu et al. (2017). For the preparation of seed cultures, ISP-2 medium was used. A 250 mL flask containing 100 mL of ISP-2 was prepared and autoclaved at 121°C for 15 min. Meanwhile, the spores of *S. yanglinensis* 3–10 were washed from an ISP-2 slant. When the medium was cooled down, it was inoculated with the spore suspension of *S. yanglinensis* 3–10 at 5 mL/flask (1 × 10^8^ spores/ml). Then, the flasks were mounted on a shaker and incubated at 28°C at 150 rpm for 24 h for the production of AFS.

### 2.5 Fermenter size and incubation time

Initially, fermentation was performed in a 5 L fermenter to optimize again some of the parameters that were already optimized during submerged fermentation in shaking flasks. After the optimization was complete, fermentation was carried out in a 30 L fermenter. Samples were taken at regular intervals, and bioassay was performed to evaluate the production of AFS by *S. yanglinensis* 3–10, and AFS yield was calculated using the standard curve.

To evaluate the best incubation time in the fermenter, fermentation was performed up to 120 h for the first three batches, and samples were taken every 12 h. The biomass was weighed, and the AFS yield was calculated according to the standard curve for each batch.

### 2.6 Fermentation runs

To determine the ideal aeration rate and agitation speed, a 5.7 L fermenter (Bioflo-IIc, New Brunswick Scientific Co., United States) with 4.5 L of production medium—as mentioned in Section 1.2—was employed. The previously mentioned inoculum was added to the fermenter at a ratio of 5% (v/v). Two six-bladed Rushton impellers (whose diameter was one-third of the tank) were used to create agitation. To stop vortex formation, four side-walled, equally spaced baffle plates were employed. A glass electrode submerged in the fermentation broth was used to test the pH. At Ingold, Leicester, United Kingdom, polarographic electrodes were used to monitor dissolved oxygen (DO). After sterilization, air-saturated medium (100%) and nitrogen-saturated medium (0%) were used for calibration (the percentage of atmospheric oxygen). When foaming started, a diluted antifoaming agent (KM-70, Hsin-Yu Co., Tokyo, Japan) was added. Throughout the investigation, the back pressure was 0.5 kg cm^2^. The agitation rates used in the trials were 150, 200, and 250 rpm. The appropriate temperature was maintained at 28 ± 2°C, and the corresponding aeration rate was varied to 0.5, 0.75, and 1.0 vvm (volume of air/volume of medium/min), respectively. Periodically, samples were taken from the fermenter, and AFS yield and antifungal activity of each batch were assessed.

The fermentation was also performed in 15 L and 30 L fermenters by using the running conditions in the 5 L fermenter (agitation speed 200 rpm, aeration rate 0.75 vvm, and starch 3% as carbon source). The representative samples were taken every 24 h up to 72 h. Biomass was weighed, and inhibition zone diameter (IZD) was determined.

### 2.7 Optimization of the carbon source

Reveromycin A and B were identified as the active compounds produced by *S. yanglinensis* 3–10 in continuation of previous published studies that involved the optimization of cultural medium, antifungal activity testing of cultural filtrate, crude extract, and volatile organic compounds against *Aspergillus flavus, Plasmodiophora brassicae, Botrytis cinerea,* and *Rhizoctonia stolonifer* (Shakeel et al., 2016; Lyu et al., 2017, 2020). To enhance the production of active compounds in fermenter, carbon source was again optimized. Only two carbon sources (starch and glucose), to which *S. yanglinensis* 3–10 respond better during shake flask fermentation, were evaluated again (Shakeel et al., 2016). Other components of the medium were the same and were already optimized during the shake flask fermentation. Cultural conditions were as follows: temperature 28°C, medium capacity 5 L, pH 6.5, aeration rate 0.75 vvm, and agitation rate 200 rpm up to 72 h.

### 2.8 Yield calculation

The IZD data were used to calculate the yield of AFS produced by *S. yanglinensis* 3–10. By using the standard growth curve of *S. yanglinensis* 3–10, a formula was derived to calculate the yield of AFS which is: Y = 11.005X+ 0.743, where Y = diameter of inhibition zone (mm) and X = yield (g/L) of AFS.

### 2.9 Statistical analysis

Data were analyzed using analysis of variance (ANOVA) in SAS software (version 9.1, SAS Institute Inc. Cary, NC, United States). Treatment means for different treatments in each experiment were separated using Fisher’s protected least significant difference (LSD) test at *p* = 0.05.

## 3 Results

### 3.1 Effect of agitation speed

**Figure 1:**
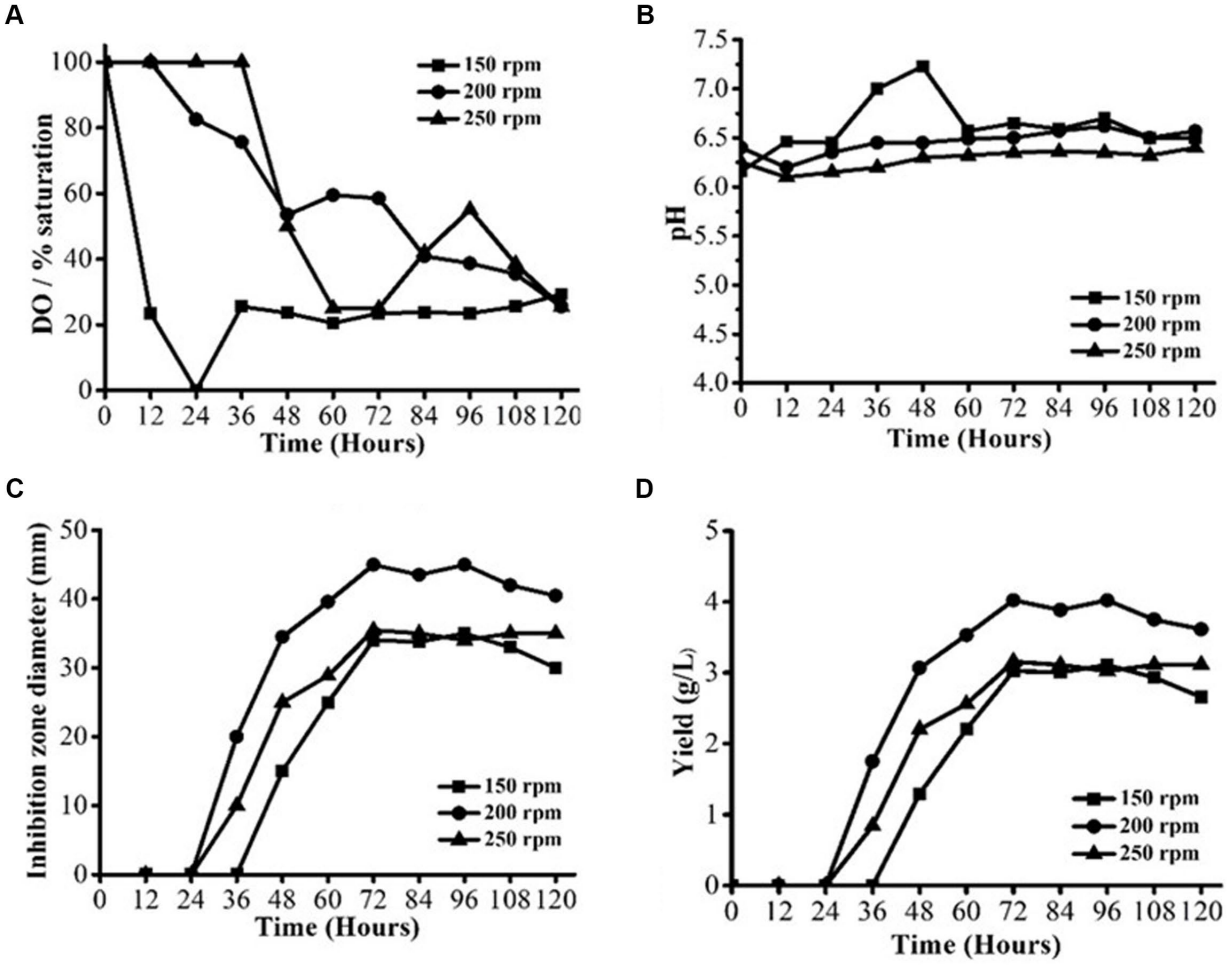
AFS production by *Streptomyces yanglinensis* 3–10 at different agitation speeds. **(A)** Dissolved oxygen (DO) saturation, **(B)** pH value, **(C)** Inhibition zone diameter, and **(D)** Yield of AFS.

Fermentation was conducted with varying agitation speeds of 150, 200, and 250 rpm, respectively, but at a constant temperature of 28 ± 2°C and aeration rate of 0.75 vvm (Figures 1A–D). For each of the three agitation levels, there were different profiles of dissolved oxygen content (Figure 1A). At 150 and 200 rpm, the DO concentration could be reliably kept above 20% saturation during the whole fermentation process. At the start of the log-growth phase, the DO concentration was noticeably lower at 150 rpm and even approached 0% saturation. Lower cell density and shorter log-growth phase at 150 rpm were the outcomes of the DO limitation. The pH profiles could be used to confirm the shift in metabolisms at 150 rpm (Figure 1B). The pH profiles at 200 and 250 rpm were not the same as the pH profile at 150 rpm. Because of the inadequate DO supply at 150 rpm, the tricarboxylic acid (TCA) cycle would not have produced much organic acid. Thus, in the cell log-growth phase at 150 rpm, the pH was greater than the pH at 200 at 250 rpm. The amount of DO and the dispersion of macromolecules in the medium increased with a higher agitation speed. Therefore, it could have had a role in the improved enzyme synthesis, and increased growth was observed in our investigation. However, cell growth and enzyme stability may be adversely affected by the shearing impact that the high agitation speed and enzyme inactivation cause in the cells. Change in profiles of DO and pH at different agitation speeds had affected AFS production by *S. yanglinensis* 3–10 (Figure 1C). The lowest antifungal activity was observed at 150 rpm while the highest antifungal activity was observed at 200 rpm. A similar trend was observed when the yield of AFS was calculated (Figure 1D). The highest yield (4.2 g/L) was observed when fermentation was performed at 200 rpm. The study indicated that 200 rpm was optimum for *S. yanglinensis* 3–10 to produce AFS.

### 3.2 Effect of the aeration rate

**Figure 2:**
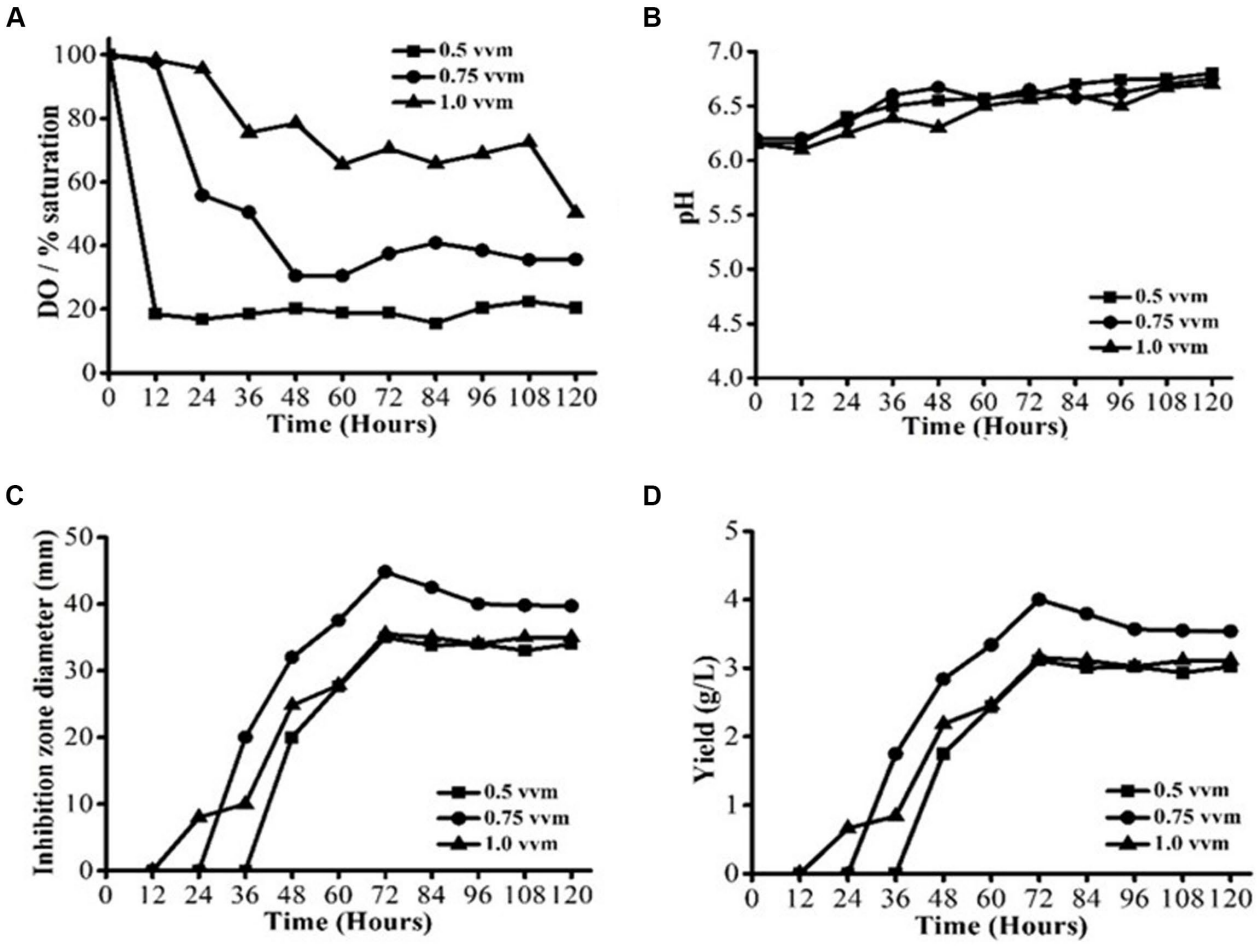
AFS production by *Streptomyces yanglinensis* 3–10 at different aeration rates. **(A)** Dissolved oxygen (DO) saturation, **(B)** pH value, **(C)** Inhibition zone diameter, and **(D)** Yield of AFS.

To examine the impact of aeration, three different aeration rates were compared: 0.5 vvm (2.5 L/min), 0.75 vvm (3.75 L/min), and 1.0 vvm (5 L/min). The operating temperature and agitation speed were kept at 28 ± 2°C and 200 rpm, respectively (Figures 2A–D). Under varying aeration rates, the profiles of DO concentrations varied significantly (Figure 2A). The DO content was mostly between 20 and 30% saturation at a low aeration rate of 0.5 vvm. Conversely, with the higher aeration rates of 0.75 and 1.0 vvm, respectively, DO concentrations were over 20 and 60% saturation. The reduced antifungal activity at the lower aeration rate of 0.5 vvm was most likely caused by DO restriction. It has been observed that in order to meet the oxygen requirement of *S. yanglinensis* 3–10 and obtain a high degree of antifungal activity, DO concentration above 20% saturation is required. The pH profiles are not significantly impacted by aeration rate, as shown in Figure 2B. This observation implied that even at the low aeration rate of 0.5 vvm, cell metabolisms remained unchanged. In contrast to the 0% concentration at 150 rpm observed in the agitation investigation, the lowest DO concentration of 20% at 0.5 vvm (Figure 2A) was greater. Consequently, *S. yanglinensis* 3–10 metabolisms were unaffected by the minimum DO concentration of 20% saturation; instead, it only had an impact on growth and AFS production. *S. yanglinensis* 3–10 growth, AFS production, and metabolisms were all impacted by the minimum DO concentration of 0%. These findings suggested that aeration might have a substantial impact on DO concentration, which would then have an impact on *S. yanglinensis* 3–10 cell proliferation, antifungal activity, and substrate utilization. At aeration rates of 0.5 and 1 vvm, there was no discernible variation in the maximal antifungal activity (Figure 2C). The AFS yield was computed using the IZD data (Figure 2D). Based on the findings of this investigation, 0.75 vvm was determined to be the ideal aeration rate because a higher level of aeration would require more power.

### 3.3 Effect of carbon source

**Figure 3:**
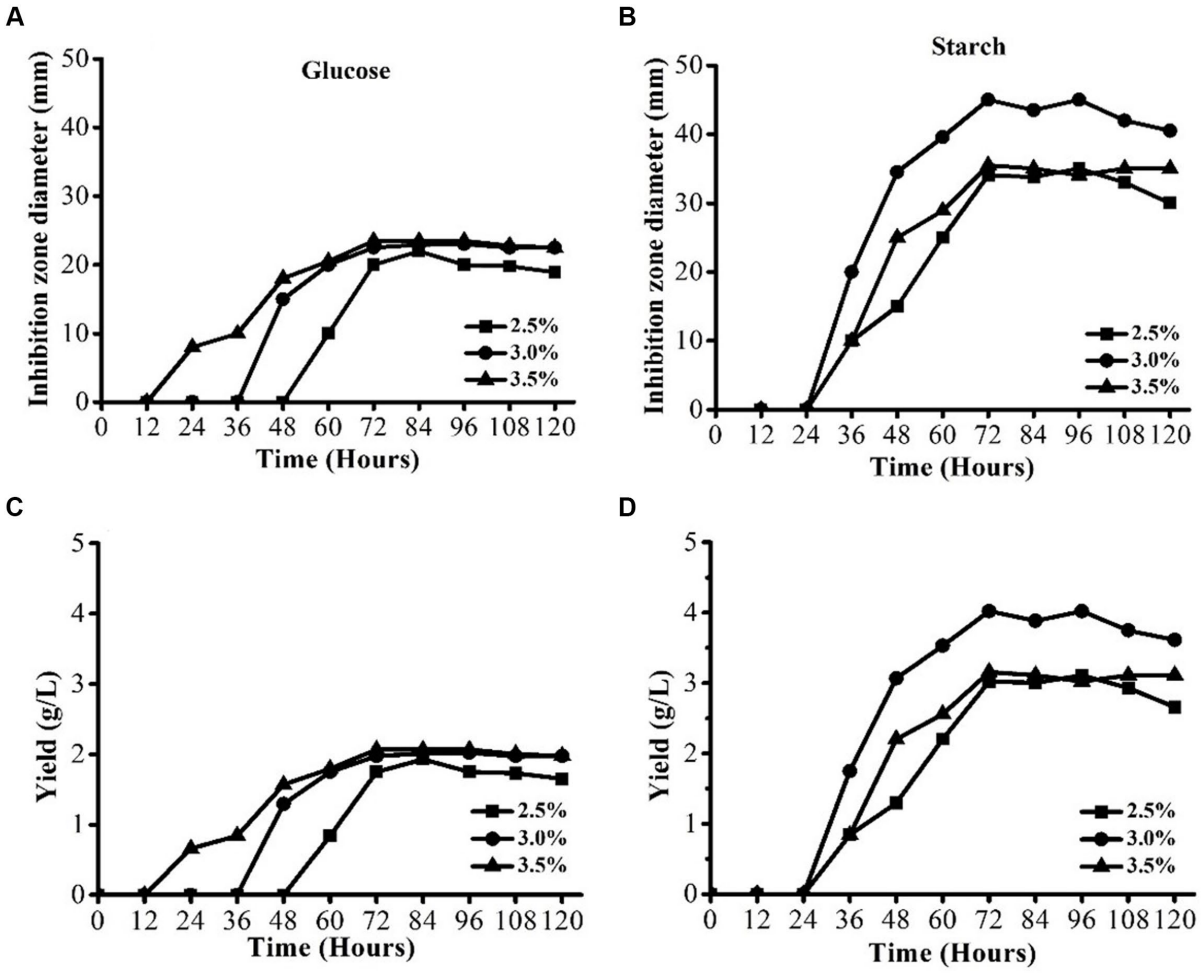
AFS production by *Streptomyces yanglinensis* 3–10 in media containing different levels of glucose and starch. **(A)** Glucose inhibition zone diameter, **(B)** Starch Inhibition zone diameter, **(C)** Yield of AFS in case of glucose, and **(D)** Yield of AFS in case of starch.

The effect of two carbon sources, namely, starch and glucose at three different levels (2.5, 3, and 3.5%) was evaluated to find out the optimum carbon source with the optimum level. The results are shown in Figures 3A,B. Figures showed that the best carbon source was starch when it was used at different levels (Figure 3A2). The highest IZD of 45 mm was achieved when 3% of starch was used. The IZDs at all levels of starch were higher than all levels of glucose. Similar results were observed regarding the AFS yield (Figure 3B). These results indicated that starch is the most suitable carbon source for *S. yanglinensis* 3–10, and these results were also in agreement with those in the shake flask fermentation results.

### 3.4 Incubation time

**Figure 4:**
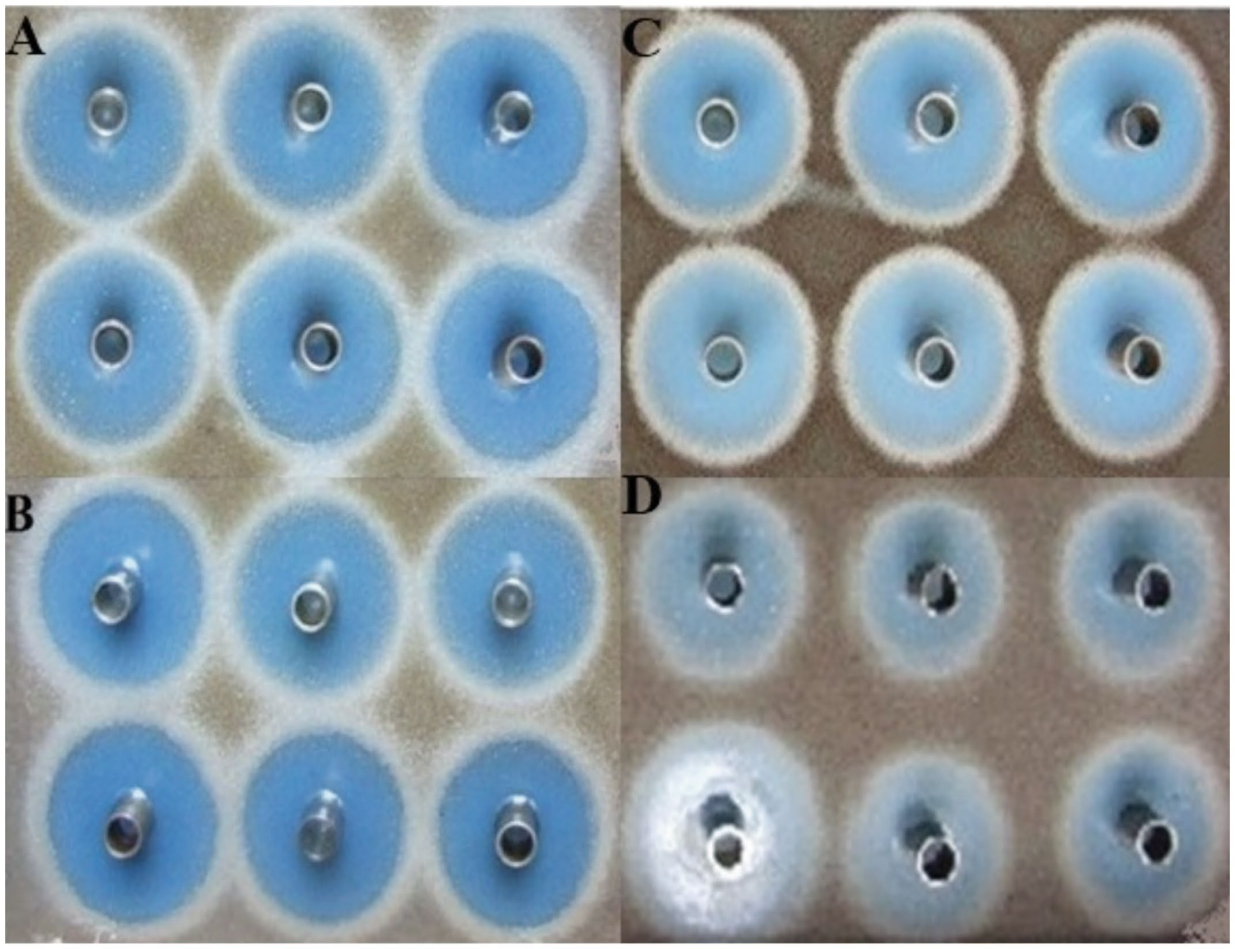
Comparison of inhibition zone produced by *Streptomyces yanglinensis* 3–10 in square-shaped plates after 72 h of fermentation. **(A–C)** 5 L, 15 L, and 30 L fermenters, respectively, whereas **(D)** denotes the control treatment.

Except for the three parameters mentioned above, the incubation time also played an important role in AFS production by *S. yanglinensis* 3–10 (Figures 4A–C). It was observed that, with the extension of the fermentation period, the antifungal activity increased and reached the peak and then declined. The maximum AFS production occurred at 72 h post-fermentation, and then, it became constant for almost the next 24 h. After 96 h of fermentation, the antifungal activity declined. The same was true when the optimization of carbon sources was carried out (Figure 3). Moreover, keeping in view all these facts, it was concluded that the optimum incubation time for *S. yanglinensis* 3–10 was from 72 h to 96 h with 72 h being the best incubation time.

### 3.5 Large-scale fermentation

The results of the antifungal activity of *S. yanglinensis* 3–10 after fermentation in 15 L and 30 L fermenters did not differ from that in the 5 L fermenter (Figure 4). The largest IZD obtained was 50 mm, which occurred in 5 L fermenter. In the case of the 15 L and 30 L fermenters, there was very little variation in IZD. However, the average IZD was 44 mm in case of the 30 L fermenter. Furthermore, it was determined that 200 rpm of agitation speed, 0.75 vvm of aeration rate, pH 6.57–6.8, and more than 20% DO would yield the best results for the large-scale batch fermentation of *S. yanglinensis* 3–10 in optimized media.

## 4 Discussion

The study delves into the impact of different operational parameters on the production of antifungal substances (AFSs) by *Streptomyces yanglinensis* 3–10. It investigates the effects of various factors such as agitation speed, aeration rate, carbon source, incubation time, dissolved oxygen (DO) concentration, pH, and temperature on the production of AFS. Several studies have been conducted based on the optimization of various cultivation and fermentation techniques to demonstrate several aspects of metabolic transition (Nieselt et al., 2010; Battke et al., 2011; Thomas et al., 2012; Wentzel et al., 2012a,b). We found that DO and pH play important roles in the production of AFS. According to Yi et al. (2014), the optimal pH for the production of antibiotics in *Streptomyces* species is close to neutral. In this study, the optimum pH for AFS production in *S. yanglinensis* 3–10 was found to be 6.5, and the findings are consistent with the study by Yi et al. (2014) and our previous study (Shakeel et al., 2016). Since it is commonly accepted that the production of AFS occurs aerobically, DO plays a crucial role.

Productivity of numerous bioactive chemicals has been discovered to be influenced by the DO concentration (Dick et al., 1994; Gavrilescu and Roman, 1994; Kempf et al., 1997). As a direct parameter of product creation, DO may have a favorable effect on the kinetics of the product at higher concentrations (Plihon et al., 1996). Additionally, if the enzymatic reaction leading to the creation of the product is highly dependent on DO, it has been proposed that DO could promote the formation of metabolites (Barberis and Segovia, 1997). A study revealed that the cultivation yield of antibiotics produced by *Streptomyces coelicolor* A3(2) was influenced by the DO levels. The highest cultivation yield was achieved. The highest cultivation yield was attained by automatically adjusting the stirrer speed to maintain an optimal level of 50% DO (Wentzel et al., 2012a). DO concentration was found to significantly affect AFS production by *S. yanglinensis* 3–10. Changes in the synthesis of bioactive compounds may arise from the impact of DO on growth rate. The minimum DO concentration required for the high growth and antifungal activity was thought to be 20% saturation. The observations of the study aligned with the conclusions drawn by Ingle and Boyer (1976) and Milner et al. (1996). They stated that there would be an instantaneous drop in AFS biosynthesis when DO dropped below 20%. Furthermore, cell respiration could switch from DO to gaseous form when DO concentration drops below a certain threshold, and these phenomena can only occur when a high aeration rate is used, and if not, linear growth could continue, signifying a combined gaseous and DO deficit.

The reduction in DO and the resulting rise in CO_2_ partial pressure might trigger a change in respiration and the initiation of new metabolisms or forms. In a study, Zhou et al. (2017) reported that the production of antiviral glycoprotein GP-1 by *S. kanasenisi* ZX01 was increased to 2.54 mg/L due to sufficient DO in 5 L fermenter, and it was attributed to oxygen transfer rate (OTR). OTR is an important factor based on aeration and agitation operations in a fermenter (Mantzouridou et al., 2002; Bandaiphet and Prasertsan, 2006; Zhou et al., 2017). The idea of optimization of aeration and agitation is valuable to enhance the yield of antimicrobial GP-1 (Zhou et al., 2017). DO has a major impact on the efficiency of aerobic fermentation mechanisms (Ravichandra et al., 2006). It is possible to enhance DO supply in the fermenter by adjusting agitation and aeration. DO can promote the growth of bacteria and the synthesis of enzymes. The volumetric mass transfer coefficient (kLa) in a fluidized-bed bioreactor indicated outstanding performance regarding adequate aeration and high production of antibiotics (Ravichandra et al., 2006).

Intense agitation has the potential to generate a large shearing impact, harming the cell and inactivating the products. Furthermore, vigorous agitation would produce a lot of agitation heat, which would increase the load of heat transfer and affect the stability of the cell and products. Actually, kLa has its limitations as well. It was discovered that after kLa reached a particular value, the oxygen saturation of the respiratory route prevented hexokinase produced by *Saccharomyces cerevisiae* from being elevated any further (Silva et al., 2001). In the current study, the highest IZD was obtained in response to using 3% starch, making starch a more effective source of carbon than glucose. Similarly, several researchers have indicated that starch is the most effective carbon source for the production of antibiotics (Ahsan et al., 2017; Aliero et al., 2017; Ganesan et al., 2017; Lyu et al., 2017; Khebizi et al., 2018; Al-Dhabi et al., 2019; Caulier et al., 2019: Wentzel et al., 2012a,b). Additionally, Aliero et al. (2017) also demonstrated that yeast extract starch broth is a successful medium to produce antimicrobial substances by the actinomycetes isolate KBMWDSb6.

In addition to pH, temperature is crucial for the ability of *Streptomyces* species to produce AFS (Aliero et al., 2017; Ganesan et al., 2017; Azish et al., 2020). A suitable drop in temperature would improve mRNA stability and extend the time for AFS production within a specific temperature range. Nevertheless, because the rate of biological reactions usually decreases with decreasing temperature, the operating temperature cannot be set too low. As a result, AFS output would decline, and the necessary fermentation time would increase at an operating temperature which is too low. Additionally, when the temperature drops too much below the surrounding air temperature, operating an industrial-scale fermenter becomes difficult. The results of this investigation indicate that *S. yanglinensis* 3–10 should be fermented at 28°C. This guarantees quick development, quick build-up of biomass, avoidance of early initiation of the metabolic transition, and effective synthesis of secondary metabolites.

## 5 Conclusion

In conclusion, the current study investigated the effects of various factors on the yield of antifungal substances (reveromycin A and B) produced by *S. yanglinensis* 3–10. Starch (3%) being the most suitable carbon source along with higher agitation speed (200 rpm), dissolved oxygen (DO) 20%, pH 6.57–6.8, and aeration rate 0.75 vvm with an optimal incubation time of 72 h led to an increased enzyme synthesis and growth, resulting in the highest antifungal activity and AFS yield. The findings provide valuable insights into optimizing the fermentation process for enhanced AFS production. Higher aeration rates resulted in an increased dissolved oxygen concentration. The optimal incubation time for AFS production was found to be between 72 and 96 h, with 72 h showing the best results. Prolonged incubation beyond 96 h resulted in a decline in antifungal activity. Large-scale batch fermentation in 15 L and 30 L fermenters did not significantly alter the antifungal activity compared with the 5 L fermenter under optimized fermentation conditions. Overall, these findings contribute to the optimization of the fermentation process for the commercial production of AFS by *S. yanglinensis* 3–10, highlighting the importance of carefully controlling agitation speed, aeration rate, carbon source, and incubation time to maximize AFS yield and quality.

## Author contributions

LY: Conceptualization, Writing – review & editing. QS: Investigation, Methodology, Writing - original draft. XX: Visualization, Writing – review & editing. LA: Data curation, Writing – review & editing. ZC: Software, Writing – review & editing. MM: Writing - original draft, Writing – review & editing. MAS: Writing – review & editing. YI: Writing – review & editing, Project administration. AK: Writing – review & editing. MKS: Finalization, Writing - review & editing. YZ: Formal analysis, Writing – review & editing. DZ: Funding acquisition, Writing – review & editing. NKA: Validation, Writing – review & editing. JW: Supervision, resources, Writing – review & editing.
